# Nephrotic syndrome induces the upregulation of cell proliferation-related genes in tubular cells in mice

**DOI:** 10.1007/s10157-024-02608-1

**Published:** 2024-12-12

**Authors:** Yuya Suzuki, Ryohei Kaseda, Yusuke Nakagawa, Hirofumi Watanabe, Tadashi Otsuka, Suguru Yamamoto, Yoshikatsu Kaneko, Shin Goto, Taiji Matsusaka, Ichiei Narita

**Affiliations:** 1 Division of Clinical Nephrology and Rheumatology, Kidney Research Center, Niigata University Graduate School of Medical and Dental Sciences, 1-757 Asahimachi, Chuo-ku, Niigata, 951-8510 Japan; 2 Institute of Medical Sciences and Department of Molecular Life Sciences, Tokai University School of Medicine, Kanagawa, Japan

**Keywords:** Proteinuria, Cell proliferation, RNA sequencing, Nep25

## Abstract

**Background:**

Massive proteinuria, dyslipidemia, and hypoalbuminemia induced by nephrotic syndrome (NS) secondarily affect tubular cells. We conducted an RNA sequencing (RNA-seq) analysis using a mouse model of focal segmental glomerulosclerosis to clarify the impact of NS on tubular cells.

**Methods:**

We used transgenic mice expressing hCD25 in podocytes (Nep25) to induce NS by injecting human CD25-specific immunotoxin (LMB2) at a dose of 0.625 ng/g body weight. Seven days after LMB2 injection, we extracted RNA from the whole kidney and conducted an RNA-seq analysis. Subsequently, we conducted multiple immunostaining and in situ hybridization (ISH) of differentially expressed genes (DEGs) to identify their locations and associated cell types. We also investigated the expression levels of DEGs in an additional mouse model of NS induced by adriamycin.

**Results:**

After NS induction, 562 upregulated and 430 downregulated DEGs were identified using RNA-seq. An enrichment analysis revealed the upregulation of cell proliferation-related genes. We observed significant upregulation of *Foxm1*, a transcription factor linked to cell proliferation. Immunostaining and ISH showed that various tubular cells expressed *Mki67* and *Foxm1* during NS development. The adriamycin-induced NS model also demonstrated the upregulation of *Mki67* and *Foxm1* in tubular cells.

**Conclusions:**

NS induced the upregulation of cell proliferation-related genes in tubular cells without detectable renal dysfunction. Our findings may contribute to understanding the pathological effects of nephrotic syndrome on tubular cells.

**Supplementary Information:**

The online version contains supplementary material available at 10.1007/s10157-024-02608-1.

## Introduction

Nephrotic syndrome (NS) is a renal disease characterized by massive proteinuria, dyslipidemia, and hypoalbuminemia. Although podocyte dysfunction is the primary cause of NS, these three symptoms are independently associated with tubular injury and chronic kidney disease. Proteinuria has various pathogenic effects on tubular cells, including inflammation, fibrosis, and apoptosis [1–4]. Lipid metabolism disorders also induce inflammation and fibrosis via lipid accumulation in tubular cells [5–7]. Additionally, severe hypoalbuminemia leads to hypovolemia and decreased renal blood flow, which can cause ischemic tubular damage and acute kidney injury [8]. The impact of nephrotic syndrome on tubular cells is complicated and still largely elusive.

Nep25 transgenic mice, which express human CD25 on podocytes, develop podocyte-specific injury and focal segmental glomerulosclerosis when injected with human CD25-specific immunotoxin (LMB2) [9]. LMB2 dose-dependently induces proteinuria and renal dysfunction, and Nep25 mice injected with a low dose of LMB2 exhibited transient NS without renal dysfunction [10]. Since a decline in the glomerular filtration rate also induces tubular injury and fibrosis, the complications of kidney dysfunction make it difficult to clarify the impacts of massive proteinuria and dyslipidemia on tubular cells. Therefore, the Nep25 mouse model is considered suitable for investigating the impact of nephrotic syndrome on tubular cells.

We aimed to investigate transcriptome changes in tubular cells in this Nep25 mouse model by performing a whole-kidney RNA sequencing (RNA-seq) analysis that primarily reflects the gene expression of proximal tubular cells [11]. Subsequently, immunostaining and in situ hybridization (ISH) were performed for differentially expressed genes (DEGs) to identify their locations and cell types. Finally, we validated the results in Nep25 mice using another mouse model of NS induced by adriamycin.

## Materials and methods

### Animals

Nep25 transgenic mice (C57 bl/6 background) that express human CD25 specifically in the podocytes [9] were maintained at the animal facility at Niigata University (Niigata, Japan). We used male Nep25 heterozygous mice. To induce proteinuria, 8- to 12-week-old mice were intravenously injected with LMB2 at a dose of 0.625 ng/g body weight. LMB2 was diluted in phosphate-buffered saline (PBS; 0.1 mL of PBS containing 0.1% bovine serum albumin; n = 3). Seven days after LMB2 injection, the mice were intraperitoneally administered a three-anesthetic mixture of medetomidine, midazolam, and butorphanol before being sacrificed. Blood and kidney samples were collected under anesthesia for further analysis, including renal pathology assessments using periodic acid-Schiff staining. Urine albumin, creatinine, blood urea nitrogen, total cholesterol, and albumin levels of the serum and urine samples were measured at Oriental Yeast Co., Ltd (Tokyo, Japan). Throughout the study period, the mice for RNA-seq analysis were maintained in metabolic cages, and urine samples were collected every 24 h. Food and water were provided ad libitum. For the quantitative reverse-transcription polymerase chain reaction (RT-PCR) analysis, we performed the same experiments as described for additional samples (n = 6) on two separate occasions, and kidney samples were collected on day 7.

To develop a mouse model of NS induced by adriamycin, 8-week-old male BALB/cJ mice were intraperitoneally injected with 8 mg/kg adriamycin (Fujifilm Wako Chemicals) (n = 8). The control group received vehicle injections only. Fourteen days after adriamycin or vehicle injection, the mice were intraperitoneally administered a three-anesthetic mixture before being sacrificed. Blood and kidney samples were collected under anesthesia for subsequent analysis.

The animal experimental protocols were approved by the Institutional Animal Care and Ethics Committee of Niigata University (approval number SA00825).

### Immunostaining

For immunohistochemical staining, 4 μm paraffin sections were heated with Antigen Retrieval Reagent (pH 6; Enzo Life Sciences, Inc.) and incubated with a rat monoclonal antibody specific to Ki-67 (catalog no.: 14–5698-82, Invitrogen) diluted in PBS. Thereafter, the sections were incubated with N-Histofine^®^ Simple Stain™ Mouse MAX PO^®^ (Rat) (Nichirei Biosciences). The target proteins were detected using ImmPACT DAB EqV (Vector Laboratories). Ki-67-positive cells were counted in 20 high-power fields per mouse. For immunofluorescence analysis, 4 μm paraffin sections were heated with the Antigen Retrieval Reagent (pH 6; Enzo Life Sciences, Inc.) and stained with a rat monoclonal antibody specific to Ki-67 (catalog no.: 14–5698-82, Invitrogen; diluted 1:400 in PBS), rabbit polyclonal antibody specific to the Na-K-Cl cotransporter (NKCC2; catalog no., ab191315, Abcam; diluted 1:400 in PBS), rabbit polyclonal antibody specific to the sodium-chloride symporter (NCC; catalog no.: AB3553, Sigma-Aldrich; diluted 1:400 in PBS), and rabbit monoclonal antibody specific to Aquaporin 2 (AQP2; catalog no.: ab199975, Abcam; diluted 1:1000 in PBS). The specific antibodies were incubated with Alexa Fluor 596-conjugated anti-rat IgG (catalog no.: A-21209, Invitrogen; diluted 1:400 in PBS) and Alexa Fluor 488-conjugated anti-rabbit IgG (catalog no.: A32790, Invitrogen; diluted 1:400 in PBS). After incubation of the secondary antibody, proximal tubules were stained by Lotus tetragonolobus lectin conjugated with FITC (Vector Laboratories). Tissue sections were mounted using ProLong Gold Antifade Mountant with DNA Stain DAPI (Invitrogen).

### ISH with immunofluorescence staining

Slides containing 4 μm paraffin sections were subjected to ISH using the RNAscope® Multiplex Fluorescent Reagent Kit version 2 (Advanced Cell Diagnostics) according to the manufacturer’s instructions. Briefly, tissue sections were incubated at 60 °C for 30 min in a HybEZTM II oven (Advanced Cell Diagnostics), pretreated with an H_2_O_2_ solution for 10 min, and boiled with RNA scope Target Retrieval for 15 min. Then, the sections were dehydrated in ethanol and incubated with Protease Plus for 30 min at 40 °C. Hybridization was performed using mRNA probe mixtures containing RNAscope^®^ Probe-Mm-*Foxm1*-C1 and RNAscope^®^ Probe-Mm-*Mki67*-C2 (Advanced Cell Diagnostics) for 2 h at 40 °C. After hybridization, the sections were incubated with the amplification reagents AMP-1, AMP-2, and AMP-3. Then, the sections were incubated with C1-horseradish peroxidase, and the signals were detected using TSA Plus Cyanine 3.5 (Akoya Biosciences). After incubation with the horseradish peroxidase blocker, C2-horseradish peroxidase incubation and detection with TSA Plus Cyanine 5 were performed as described. After the RNAscope protocol, the sections were subjected to heat-mediated antigen retrieval and stained with a rabbit polyclonal antibody specific to NKCC2 (catalog no.: ab191315, Abcam; diluted 1:400 in PBS), rabbit polyclonal antibody specific to NCC (catalog no.: AB3553, Sigma-Aldrich; diluted 1:400 in PBS), rabbit monoclonal antibody specific to AQP2 (catalog no.: ab199975, Abcam; diluted 1:1000 in PBS), and FITC-conjugated Lotus tetragonolobus lectin (Vector Laboratories) to identify the type of tubules. Then, these antibodies were incubated with Alexa Fluor 488-conjugated anti-rabbit IgG (Abcam). Finally, the slides were mounted using ProLong Gold Antifade Mountant with DAPI DNA Stain (Invitrogen).

### Real-time PCR

Kidney RNA was extracted using an RNeasy Universal Mini Kit (Qiagen, Hilden, Germany). A quantitative real-time PCR was conducted using the One Step SYBR PrimeScript Plus RT-PCR Kit (Takara Bio) according to the manufacturer’s protocol. *Gapdh* and *18S rRNA* were used as the reference genes. The primers used in this study are listed in Online Resource 1.

### RNA sequencing

Kidney RNA was extracted using an RNeasy Universal Mini Kit (Qiagen). The RNA integrity number was analyzed using microcapillary electrophoresis (TapeStation 4200; Agilent Technologies), and all RNA samples had an RNA integrity number > 8. Total RNA samples were subjected to stranded library preparation with polyA selection, multiplexing, and sequencing, which were performed by Genewiz (South Plainfield, NJ, USA). Sequencing was performed on the Illumina NovaSeq platform using a 2- × 150-bp paired-end configuration with 6.0 Gb of data per sample and a total of at least 36.0 Gb. The quality of the sequencing data was assessed using FastQC (version 0.10.1), and data filtering was performed using Cutadapt (version 1.9.1). Hisat2 (version 2.0.1) was used to align the data with the reference genome Musculus Ensembl_GRCm38.101. Read counts for each gene were obtained using high-throughput sequencing (version 0.6.1). A differential expression analysis was performed using the DESeq2 package (version 1.4.1) in R software (version 4.3.0). Statistical significance was set at P < 0.05. For the Kyoto Encyclopedia of Genes and Genomes (KEGG) enrichment analysis of DEGs, we used the clusterProfiler R package (version 4.8.1). Data were visualized using the ggplot2 R package (version 3.4.2).

### Data availability

The RNA-seq datasets used in this study are available in the DDBJ Sequence Read Archive (accession numbers: DRR492957-DRR492962).

### Statistical analysis

GraphPad Prism version 9.2.0 (GraphPad Software) was used for statistical analyses. All normally distributed data are shown as the mean ± standard deviation, whereas non-normally distributed data are shown as the median (interquartile range). Unpaired Student’s or Welch’s t-tests were used for continuous variables with normal distribution, whereas the Wilcoxon signed-rank test was used for continuous variables with non-normal distribution. All reported P values were two-sided, and P < 0.05 was considered statistically significant.

## Results

### Induction of NS for RNA-seq in Nep25 mice

**Fig. 1:**
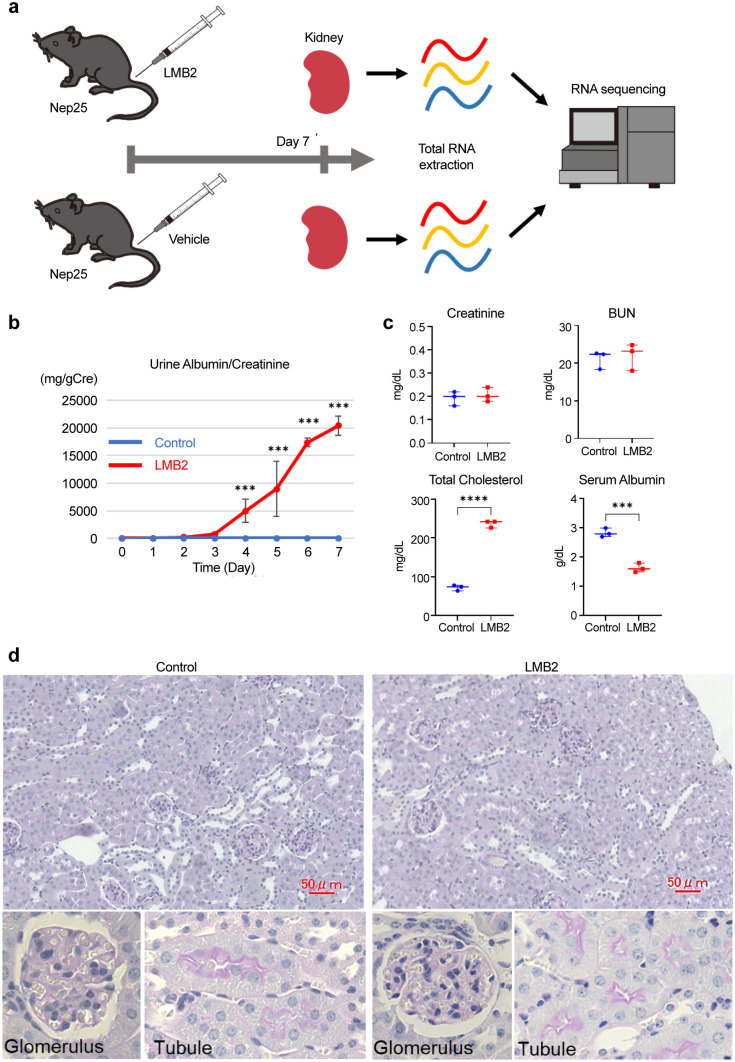
Induction of nephrotic syndrome for the RNA-sequencing (RNA-seq) analysis. **a** Schematic representation of the RNA-seq protocol for Nep25 mice. **b** Urine albumin levels after LMB2 or vehicle injection (*n* = 3). Error bars represent the standard deviation. ***P < 0.001 (unpaired t-test). **c** Serum creatinine, blood urea nitrogen (BUN), total cholesterol, and albumin levels 7 days after LMB2 injection (n = 3). ***P < 0.001 and ****P < 0.0001 (unpaired t-test). **d** Periodic acid-Schiff (PAS) staining of 4 μm paraffin sections of the kidney

A schematic representation of the RNA-seq in Nep 25 mice is shown in Fig. 1a. After the injection of LMB2 or vehicle, Nep25 mice had proteinuria from day 4, which escalated to massive proteinuria, hypoalbuminemia, and hypercholesterolemia by day 7 (Fig. 1b, c). Body weight, food intake, and fluid intake did not differ significantly between groups (Online Resource 2). Blood urea nitrogen and serum creatinine levels did not increase substantially in Nep25 mice injected with LMB2 on day 7 (Fig. 1c). Moreover, periodic acid-Schiff staining did not reveal any notable pathological changes in the kidney tissue (Fig. 1d). The total RNA extracted from whole kidneys on day 7 was subjected to RNA-seq.

### RNA-seq analysis revealed upregulation of cell proliferation-related genes and downregulation of fatty acid oxidation-related genes

**Fig. 2:**
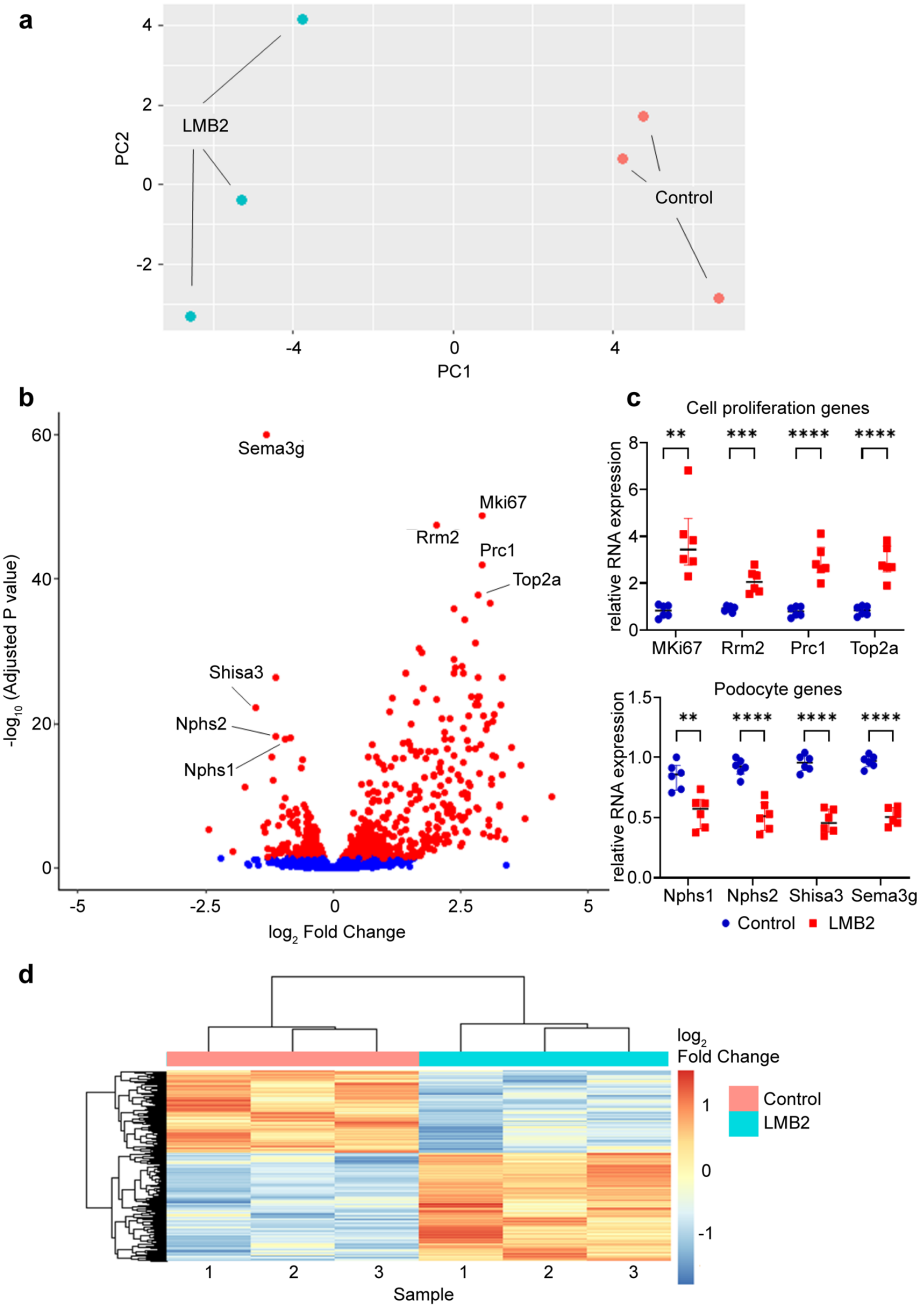
Nep25 mice with nephrotic syndrome represent distinct transcriptomic profiles. **a** Principal component (PC) analysis plot comparing the control group and LMB2 group (n = 3). **b** Volcano plot representing all expressed genes. The log2 fold change indicates the relative mRNA expression of the LMB2 group compared to that of the control group. **c** Quantitative reverse-transcription polymerase chain reaction analysis of differentially expressed genes (DEGs) shown in the volcano plot (n = 6). **P < 0.01, ***P < 0.001, and ****P < 0.0001 (unpaired t-test). **d** Heatmap generated using the DEGs

**Fig. 3:**
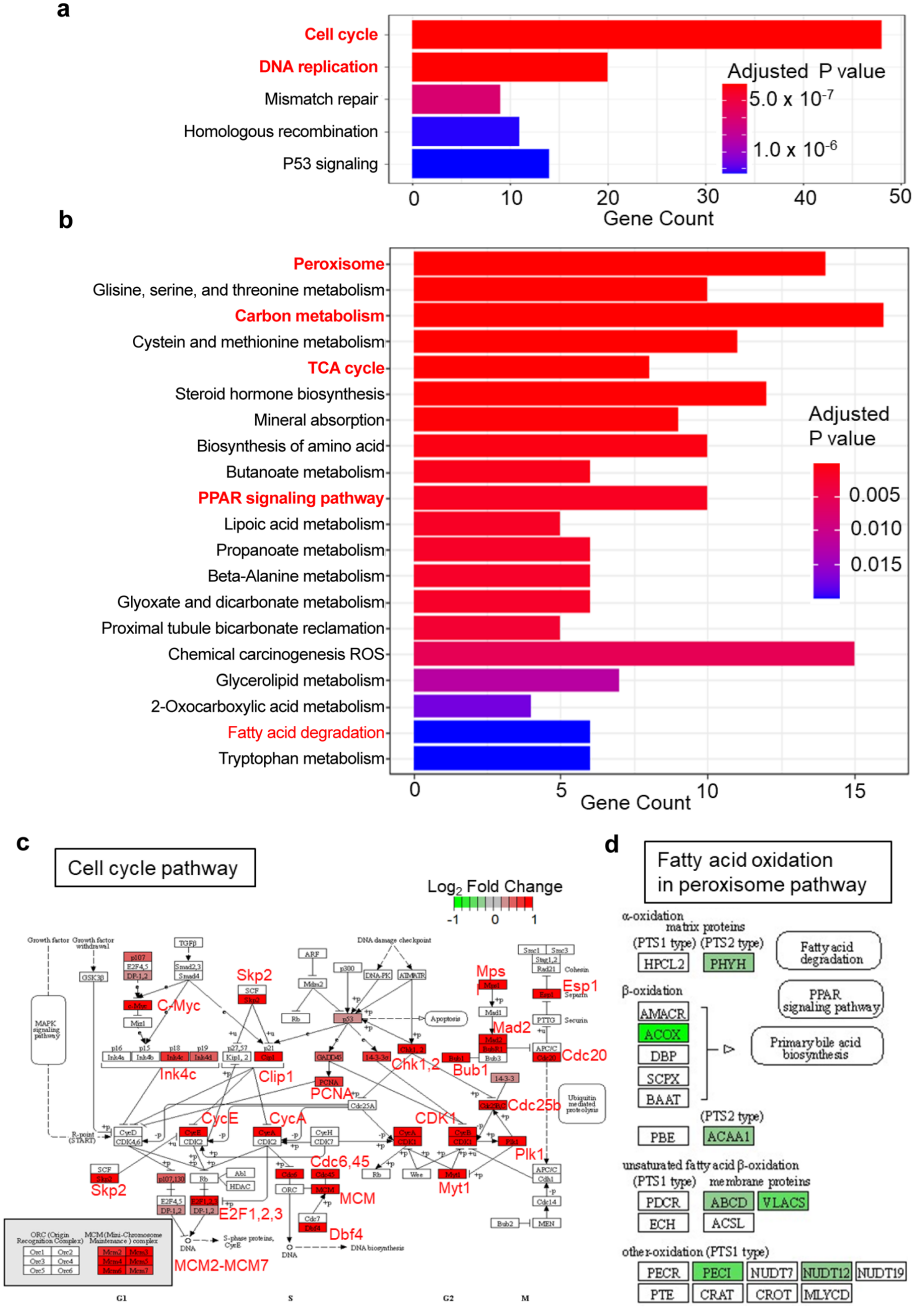
Enrichment analysis of differentially expressed genes (DEGs) demonstrates the upregulation of cell proliferation-related genes and downregulation of FAO-related genes. **a** Kyoto Encyclopedia of Genes and Genomes (KEGG) enrichment analysis of significantly upregulated genes in the LMB2 group. The bar plot was generated by using clusterProfiler. **b** KEGG enrichment analysis of significantly downregulated genes in the LMB2 group. **c** Cell cycle pathway map (mmu4110) of the KEGG database illustrated by clusterProfiler. Upregulated genes in the LMB2 group compared to the control group are depicted in red. **d** Fatty acid oxidation (FAO) in the peroxisome pathway map (mmu04146) of the KEGG database illustrated by clusterProfiler. Downregulated genes in the LMB2 group compared to the control group are depicted in green. *PPAR* peroxisome proliferator-activated receptor, *ROS* reactive oxygen species, *TCA* tricarboxylic acid

The principal component analysis plot demonstrated distinct trends in gene expression between groups (Fig. 2a). The volcano plot provided a comprehensive overview of the differential expression of mRNAs (Fig. 2b). Specifically, 562 genes were upregulated, and 430 genes were downregulated in the LMB2 group compared to the control group. The highly upregulated genes included *Mki67*, *Rrm2*, *Prc1*, and *Top2a*, which are associated with cell proliferation. The downregulated genes included *Sema3g*, *Shisa3*, *Nphs1*, and *Nphs2*, all of which are expressed in podocytes [12–14]. These results were confirmed using a quantitative RT-PCR (Fig. 2c). A heat map generated using the DEGs is presented in Fig. 2d. The KEGG enrichment analysis highlighted the remarkable upregulation of genes involved in the cell cycle and DNA replication pathways (Fig. 3a, c). In contrast, genes associated with fatty acid oxidation (FAO) in the peroxisome and the tricarboxylic acid cycle were significantly enriched among the downregulated genes (Fig. 3b, d).

### Cell proliferation-related genes including Foxm1 were upregulated in various tubular cells

**Fig. 4:**
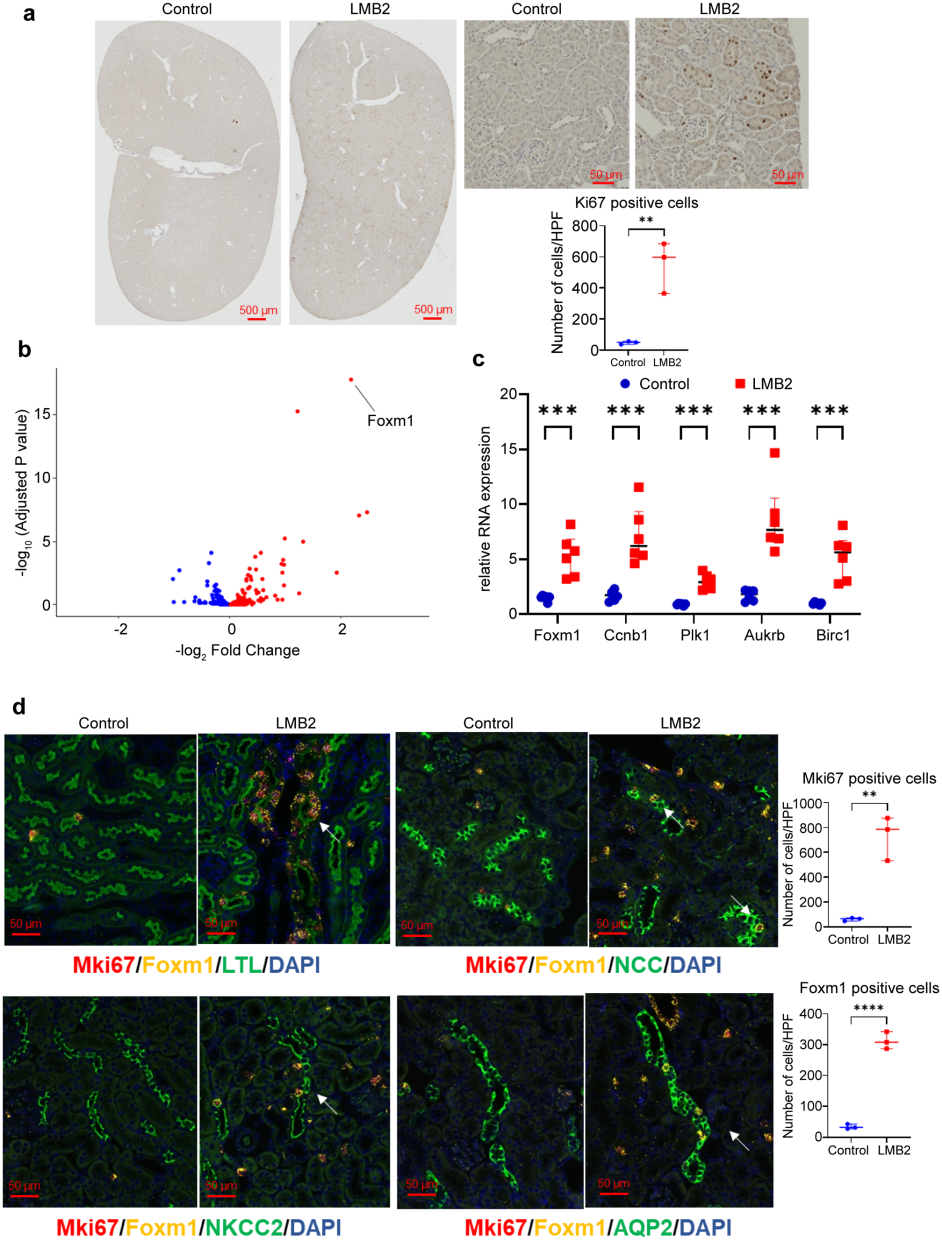
*Mki67* and *Foxm1* were upregulated in various tubular cells. **a** Ki-67 immunostaining performed on 4 μm paraffin kidney sections. The number of Ki-67-positive cells was assessed in a minimum of 20 randomly selected high-power fields per animal (n = 3). **P < 0.01 (unpaired t-test). **b** Volcano plot of genes that have the ontology term of DNA-binding transcription factor activity (GO:0003700). **c** mRNA expression of *Foxm1* and its downstream targets analyzed by the quantitative reverse-transcription polymerase chain reaction (n = 6). ***P < 0.001 (unpaired t-test). **d** Multiple in situ hybridization (ISH) procedures performed for *Foxm1* and *Mki67*. The sections were subjected to immunostaining of tubular markers after ISH. *MKi67*-positive cells and *Foxm1*-positive cells were counted in a minimum of 20 randomly selected high-power fields per animal (n = 3). **P < 0.01 and ****P < 0.0001 (unpaired t-test). Lotus tetragonolobus lectin (LTL), NKCC2, NCC, and AQP2 were utilized as markers for the proximal tubule, thick ascending limb, distal tubule, and collecting duct, respectively

To identify the cell type and localization of cell proliferation, we performed immunohistochemistry and immunofluorescence staining of Ki-67 in kidney tissue (Fig. 4a). Ki-67 was primarily expressed in tubular cells, and the number of Ki-67-positive cells increased in kidney samples with LMB2. Multiple immunostaining procedures revealed that Ki-67 was expressed in various tubular cells, including those in the Lotus tetragonolobus lectin-positive proximal tubule, NKCC2-positive thick ascending limb, NCC-positive distal tubule, and AQP2-positive collecting duct (Online Resource 3). To investigate the transcription factors involved in cell proliferation, we extracted genes with the ontology term of DNA-binding transcription factor activity (GO:0003700) from our RNA-seq data and illustrated a volcano plot (Fig. 4b). Consequently, the transcription factor *Foxm1*, which promotes cell proliferation [15–17], was prominently upregulated. Quantitative RT-PCR confirmed the upregulation of *Foxm1* and its downstream targets (Fig. 4c). To investigate the localization of *Foxm1*, we performed multiple ISH assays for *Mki67* and *Foxm1* along with immunostaining for tubular markers (Fig. 4d). *Foxm1* and *Mki67* mRNA were co-expressed in various tubular cells, including the proximal tubule, thick ascending limb, distal tubule, and collecting duct.

### A mouse model of adriamycin-induced NS also demonstrated the upregulation of Foxm1 and Mki67

**Fig. 5:**
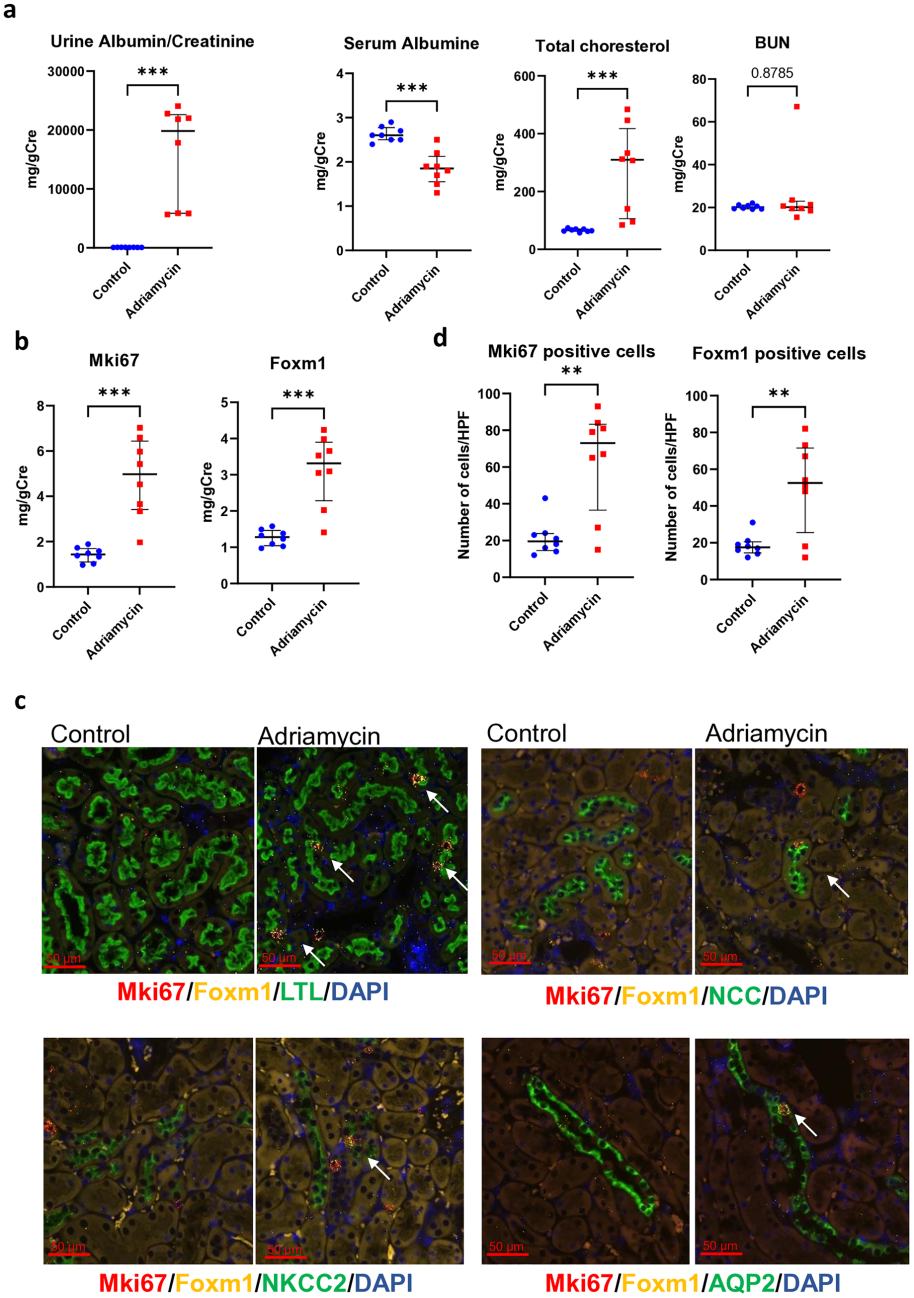
*Mki67* and *Foxm1* were upregulated in the adriamycin-induced nephrotic syndrome model. **a** Urine albumin, serum albumin, total cholesterol, and blood urea nitrogen (BUN) levels 14 days after adriamycin injection (n = 8). ***P < 0.001 (unpaired t-test). **b** mRNA expression of *Mki67* and *Foxm1* analyzed by quantitative reverse-transcription polymerase chain reaction (n = 8). **c** Multiple in situ hybridization (ISH) procedures performed for *Foxm1* and *Mki67*. The sections were subjected to immunostaining for tubular markers after ISH. ***P < 0.001 (unpaired t-test). Lotus tetragonolobus lectin (LTL), NKCC2, NCC, and AQP2 were utilized as markers for the proximal tubule, thick ascending limb, distal tubule, and collecting duct, respectively. **d** The number of *Mki67*-positive cells and *Foxm1*-positive cells was counted in a minimum of 20 randomly selected high-power fields per animal (n = 3). **P < 0.01 (unpaired t-test)

To validate our findings in NEP25 mice, we utilized a mouse model of adriamycin-induced NS and investigated the expression levels of cell proliferation-related genes. Fourteen days after an 8 mg/kg adriamycin injection, the mice developed massive proteinuria, hypoalbuminemia, and hypercholesterolemia without significant elevation of blood urea nitrogen (Fig. 5a). Periodic acid-Schiff staining did not reveal any notable pathological changes in the kidney tissue (Online Resource 4). mRNA expression of *Foxm1* and *Mki67* also significantly increased in this nephrotic syndrome model (Fig. 5b). Multiple ISH assays for *Foxm1* and *Mki67* demonstrated the upregulation of these genes in tubular cells (Fig. 5c, d), which was consistent with the results observed in NEP25 mice.

## Discussion

This study demonstrated changes in the kidney transcriptome in a mouse model of NS. We identified significant upregulation of cell proliferation-related genes and downregulation of FAO-related genes. ISH of *Mki67* and *Foxm1* indicated cell proliferation in various tubular cells. We confirmed that these results are consistent with those in a mouse model of adriamycin-induced NS.

We found that cell proliferation-related genes were upregulated in tubular cells during the development of NS. Matsusaka et al. previously reported that Nep25 mice with glomerular sclerosis and tubular injury injected with LMB2 at a dose of 5 ng/g body weight showed proliferation of tubular and glomerular cells [9]. Nep25 mice injected with a much lower LMB2 dose of 0.625 ng/g body weight in our study also showed tubular cell proliferation without detectable renal histological damage. Additionally, the adriamycin-induced NS model mice demonstrated similar results. These findings indicate that NS may induce tubular cell proliferation prior to the development of detectable renal dysfunction.

When focusing on transcription factors, *Foxm1*, which has been reported to promote kidney cell proliferation after ischemia–reperfusion [18, 19], was upregulated in tubular cells. The RNA-seq analysis of three-dimensional cultured human proximal tubule cells demonstrated that the addition of human serum to the medium induced the expression of cell proliferation genes, including *Foxm1* [20]. As such, the upregulation of proliferation-related genes may be triggered by the leakage of serum components from the glomerulus. Another possible explanation is that microtubular injury induced by hemodynamic disorders caused by hypoalbuminemia or lipotoxicity caused by dyslipidemia may result in tubular cell recovery and proliferation. Because a combination of several factors may contribute to tubular proliferation, further studies are needed to clarify the underlying mechanisms. Additionally, our study revealed that *Foxm1*- and *Mki67*-positive cells were present not only in the proximal tubule but also in the thick ascending limb, distal tubule, and collecting duct. This result indicated that NS development can influence various types of tubular cells, leading to induced proliferation.

The role of upregulated cell proliferation-related genes in tubular cells is controversial. In the ischemia–reperfusion model, tubular cell proliferation plays a crucial role in the recovery of damaged kidney tissue [19]. As such, the upregulation of *Mki67* and *Foxm1* may indicate the recovery process of tubular cells from micro-injury induced by nephrotic syndrome. Conversely, several studies have reported the negative effects of upregulated Foxm1, which promotes fibrosis in kidney [21, 22]. As we investigated an NS model without renal dysfunction or renal fibrosis, further studies are needed to elucidate the role of tubular cell proliferation in the pathophysiology of NS.

The RNA-seq analysis also revealed the downregulation of genes in the FAO pathway. Renal tubular cells are highly dependent on FAO for ATP synthesis [23], and dysregulation of this pathway induces mitochondrial and cellular damage [7]. Impaired FAO has been observed in patients with chronic kidney disease [5], and renal ischemia or drug-induced tubular injury causes the downregulation of FAO [24, 25]. In this study, although the direct cause of this metabolic change could not be determined, microtubular injury caused by proteinuria, dyslipidemia, or hemodynamic disorders attributable to hypoalbuminemia may have affected FAO. Our results indicate that downregulation of FAO may occur before the elevation of serum creatinine and blood urea nitrogen levels with NS.

Several limitations must be considered when interpreting these results. First, although we demonstrated the changes in mRNA and protein expression in nephrotic syndrome, the specific factors responsible for upregulating cell proliferation-related genes, including *Foxm1*, were not clarified in this study. Further research is necessary to elucidate the cause-and-effect relationship between nephrotic syndrome and cell proliferation. Second, despite several hypotheses as mentioned above, the functions of upregulated cell proliferation and downregulated FAO in nephrotic syndrome remain unknown. Additional studies are required to elucidate the roles of these changes in the pathophysiology of nephrotic syndrome.

In conclusion, this study demonstrated transcriptome changes induced by NS. We revealed that cell proliferation-related genes are upregulated in tubular cells without detectable renal dysfunction in NS. Our findings may provide valuable insights into understanding the impact of nephrotic syndrome on tubular cells.

## Supplementary Information

Below is the link to the electronic supplementary material.

Supplementary file1 (PDF 606 KB)

## Data Availability

The RNA-seq datasets used in this study are available in the DDBJ Sequence Read Archive (https://www.ddbj.nig.ac.jp/dra/index-e.html; accession numbers: DRR492957-DRR492962).
