# Supplementary material for: Nephrotic syndrome induces the upregulation of cell proliferation-related genes in tubular cells in mice
Source: Clin Exp Nephrol. 2024 Dec 12;29(4):393–404. doi: 10.1007/s10157-024-02608-1 (PMC11937209; doi:10.1007/s10157-024-02608-1)
Supplement: Supplementary file 1 — Supplementary file1 (PDF 606 KB) [file 10157_2024_2608_MOESM1_ESM.pdf]

Supplementary Materials

Clinical and Experimental Nephrology

Nephrotic syndrome induces tubular cell proliferation and suppresses renal fatty acid oxidation

Yuya Suzuki[1], Ryohei Kaseda[1*], Yusuke Nakagawa[1], Hirofumi Watanabe[1], Tadashi Otsuka[1], Suguru Yamamoto[1], Yoshikatsu Kaneko[1], Shin Goto[1], Taiji Matsusaka[2], Ichiei Narita[1]

[1]Division of Clinical Nephrology and Rheumatology, Kidney Research Center, Niigata University Graduate School of Medical and Dental Sciences, Niigata, Japan

[2]Institute of Medical Sciences and Department of Molecular Life Sciences, Tokai University School of Medicine, Kanagawa, Japan

***Corresponding Author:** Ryohei Kaseda

E-mail: ryoheik@med.niigata-u.ac.jp

Online Resource 1. The primers used in RT-qPCR

Gene name	Sense (5’-3’)	Antisense (3′-5′)
Gapdh	AGGTCGGTGTGAACGGATTTG	GGGGTCGTTGATGGCAACA
18S rRNA	GGACCAGAGCGAAAGCATTTGCC	TCAATCTCGGGTGGCTGAACGC
Mki67	GAAGTCTCTTGGCACTCACA	GCGTCTTTGATCATTTGTCCTC
Foxm1	GGACATCTACACTTGGATTGAGG	TGTCATGGAGAGAAAGGTTGTG
Mcm2	CCGTTCCAAGGATGCCATTCTC	TGGAAAGCCGTTGGCGGTGTTA
Ccnb1	TGAGCCTGAACCTGAACTTG	ACATCAGAGAAAGCCTGACAC
Plk1	ACCTACCTCCGGATCAAGAAA	AGAACTCGTCATTGAGCAACTC
Aurkb	TGCAGGGAGAACTGAAGATTG	CGATGCACCATAGATCTACCATT
Birc1	GGAATTGGAAGGCTGGGAA	CCATCTGCTTCTTGACAGTGA

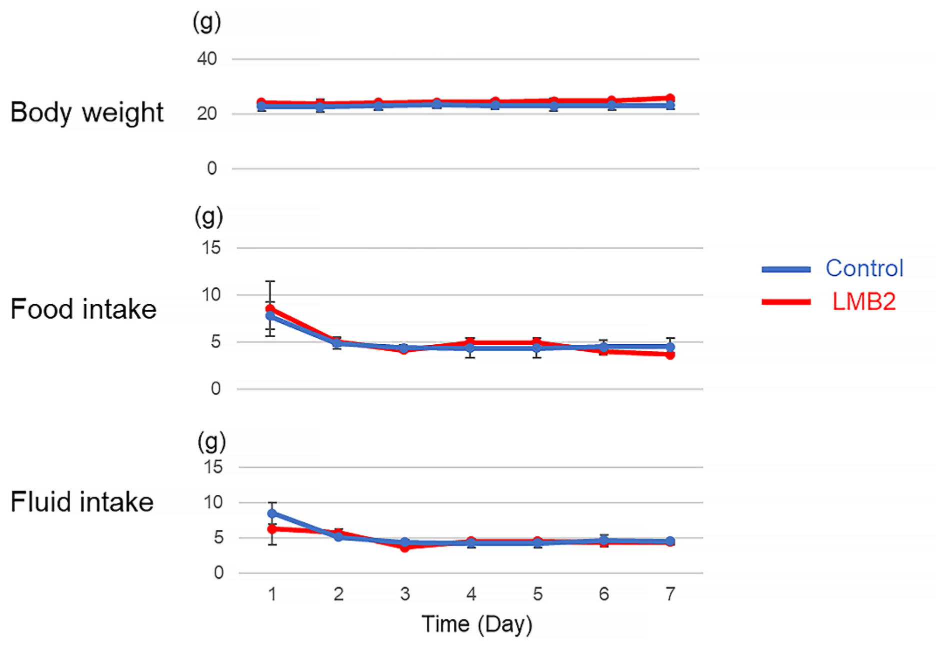

Online Resource 2. Body weight and food and water intake after LMB2 or vehicle injection (n = 3)

Error bars represent the standard deviation

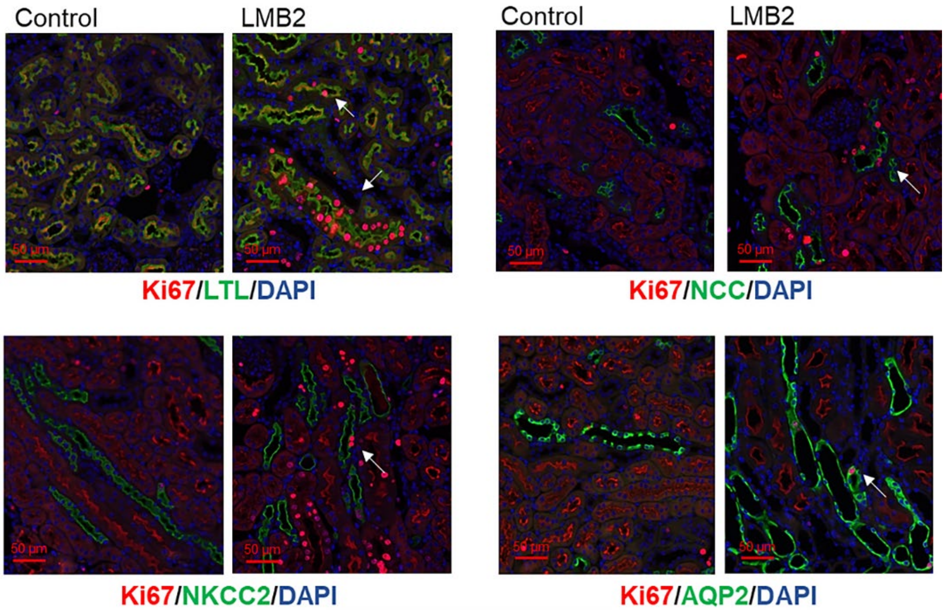

Online Resource 3. Multiple immunostaining of Ki-67 and tubular markers

Lotus tetragonolobus lectin (LTL), NKCC2, NCC, and AQP2 were utilized as markers for the proximal tubule, thick ascending limb, distal tubule, and collecting duct, respectively

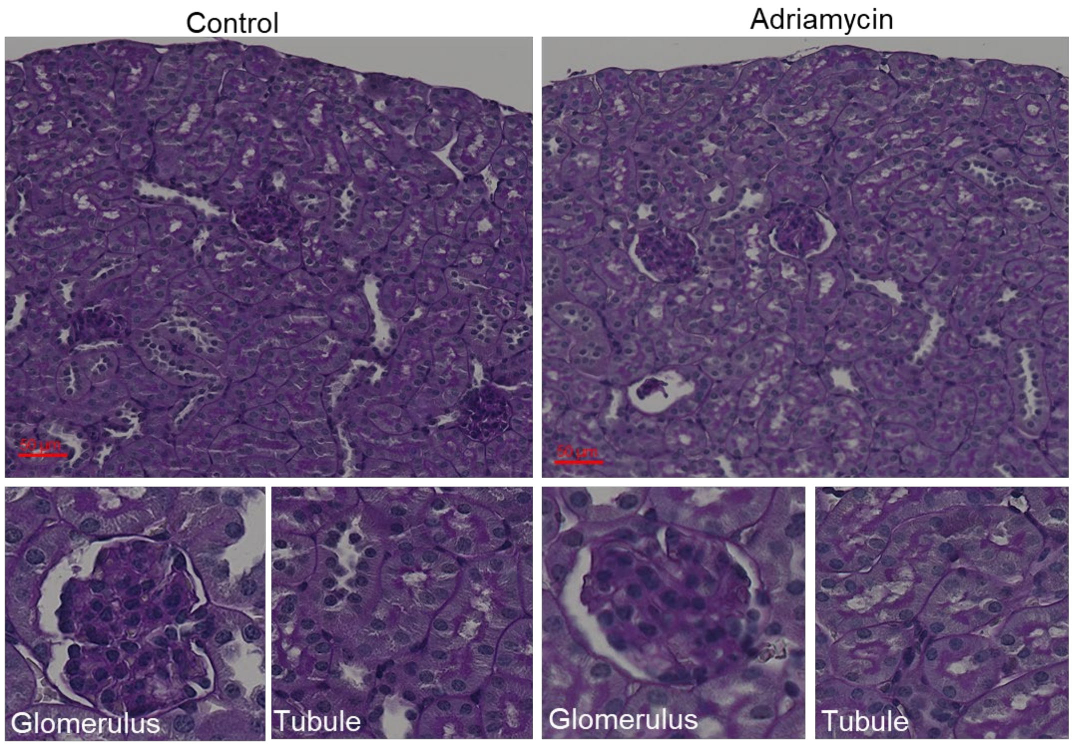

Online Resource 4. Periodic acid-Schiff staining of 4 µm paraffin sections of the kidney following Adriamycin injection
